# A Novel Exploratory Graph-Based Analytical Tool for Functional Near-Infrared Spectroscopy in Naturalistic Experiments: An Illustrative Application in Typically Developing Children

**DOI:** 10.3390/brainsci13060905

**Published:** 2023-06-03

**Authors:** João Ricardo Sato, Tiago Duarte Pereira, Clarice Maria de Lucena Martins, Thaynã Alves Bezerra, Maria Eduarda Queiroz, Larissa Pereira Costa, Suellen Marinho Andrade, Claudinei Eduardo Biazoli

**Affiliations:** 1 Center of Mathematics, Computing, and Cognition, Federal University of ABC, São Bernardo do Campo 09606-045, SP, Brazil; 2 Big Data, Hospital Israelita Albert Einstein, São Paulo 05652-900, SP, Brazil; 3 Department of Physical Education, Federal University of Paraiba, João Pessoa 58051-900, PB, Brazil; 4 Laboratory for Integrative and Translational Research in Population Health, Research Centre of Physical Activity, Health and Leisure, Faculty of Sports, University of Porto, 96810-012 Porto, Portugal; 5 Department of Physical Education, Regional University of Cariri, 63105-010 Crato, CE, Brazil; 6 Laboratory of Aging and Neuroscience Studies, Department of Physical Therapy, Health Sciences Center, Federal University of Paraíba, João Pessoa 05652-000, PB, Brazil; 7 Department of Biological and Experimental Psychology, Queen Mary University of London, London E1 4NS, UK

**Keywords:** naturalistic stimuli, functional neurodevelopment, graph-centrality, graph theory

## Abstract

Naturalistic paradigms are being increasingly applied to investigate human brain function. Compared with resting-state and task-based paradigms in neuroimaging, naturalistic stimuli and situations can be potentially more readily translated to daily-life applications. Among neuroimaging modalities, functional near-infrared spectroscopy (fNIRS) is particularly suitable for naturalistic investigations and applications. However, specific and tailored statistical analysis to interrogate brain function using naturalistic fNIRS is warranted. Here, we describe an exploratory graph-centrality-based approach to investigating participants’ spatiotemporal similarities from the fNIRS signal. We illustrate the usefulness of our approach in a sample of typically developing children (10 males and 9 females; mean age of 5.2 years old; sd = 0.78) while they watch the Inscapes movie designed for neuroimaging acquisition. A node in the left dorsal prefrontal cortex presented similar responses across children, and those fNIRS responses were in line with scene transitions in the movie stimulus. Our results suggest the feasibility of applying centrality graph-based measures to investigate brain function in naturalistic fNIRS during development.

## 1. Introduction

Naturalistic stimuli and situations are being increasingly adopted and investigated in neuroimaging [1,2,3,4]. Naturalistic paradigms are thought to improve the external validity and applicability of neuroimaging results [5]. Particularly, functional near-infrared spectroscopy (fNIRS) has been successfully used to probe human cortical function in ecologically realistic situations that are not possible using functional magnetic resonance imaging (fMRI), positron emission tomography (PET), or magnetoencephalography (MEG) [6]. However, proper statistical analysis strategies and frameworks suitable to interrogate fNIRS signal dynamics in naturalistic paradigms are necessary.

Unlike other common neuroimaging techniques (e.g., fMRI, PET, and MEG), the fNIRS signal can be acquired in a wide variety of environments. In addition, the portability of the acquisition equipment and the relative robustness of the methodology to motion artifacts allow subjects to move freely during data acquisition.

Usually, fNIRS acquisition involves multiple channels, and a channel-wise analysis strategy is applied. This approach limits statistical power since as many null hypothesis tests as channels are necessary. In these analyses, even adjusting for multiple comparisons might be insufficient to investigate associations between fNIRS signal and behavior, and massive sample sizes would be necessary [7]. Paradoxically, having more fNIRS channels available and thus more cortical spatial information sampled leads to decreased statistical power to reject the null hypothesis due to multiple testing corrections.

In the case of naturalistic stimuli, given that the fNIRS signal temporal sampling rate is in the order of tens of Hertz, the number of time-points available is an order of magnitude higher than typical fMRI acquisitions. A typical fNIRS acquisition at 7 Hz for 5 min yields 2100 time points. As an example, a 20-long-range channel-wise analysis for each time point would lead to 20 times 2100 = 42,000 multiple comparisons, which results in virtually no statistical power to detect any effect. Even worse, the probability of detecting a true effect would likely be less than a Type I error, as discussed by Ioannidis [8]. Additionally, naturalistic stimuli are usually not reducible to a block or event-related design structure. Hence, the conventional General Linear Model (GLM) approach, in which the hemodynamic signals are considered dependent variables and the experimental design is convoluted by a hemodynamic response function as the independent variable, cannot be applied once the design matrix is not specified.

Preliminary studies have shown that the use of naturalistic stimuli (e.g., movies) enhances the reproducibility of functional connectivity and large-scale functional network findings [9,10,11]. In this scenario, recent modeling methods and complexity-based analysis have been suggested to address the limitations of the classical GLM-based analysis for non-structured paradigms [12,13].

Given these challenges related to the nature of fNIRS hemodynamic signals in naturalistic situations, in many cases, it is not possible to define a priori the time points of interest. Thus, our main question in the current study is: how could we identify the time points when inter-individually consistent hemodynamic responses in relation to naturalistic stimuli happen? Identifying these most inter-individually consistent time points, we mapped the brain regions where these relevant hemodynamic responses occur. The intuitive idea of our proposed approach is to first, based on a single model and thus avoiding the multiple comparisons problem, statistically test whether the stimulus elicits a similar hemodynamic state pattern among the participants at at least one time point and channel. Then, if the null hypothesis is rejected, we conduct post-hoc exploratory analyses to further understand which time points and channels are mostly contributing to the results [6].

To carry out this analysis, we defined a “relevant” time point as a temporal frame in which the presented stimulus induces a more similar hemodynamic state pattern considering all channels across individuals. Hence, in this exploratory pilot study, we propose a novel analytical approach for finding a consistent hemodynamic response across participants in naturalistic fNIRS. A new regression method based on graph centrality analyses and bootstrapping has been developed for this purpose. Finally, we illustrated this new methodology by applying it to analyze fNIRS data from children watching an abstract and neuroimaging-tuned movie stimulus, i.e., the Inscapes movie [14].

## 2. Material and Methods

This study was approved by the Ethics Committee of the Universidade Federal da Paraíba (266391198000005188). All parents or legal guardians of the children provided informed, signed consent for participation in this study, and no financial compensation was provided to the participants.

### 2.1. Participants

Nineteen children (10 males and 9 females; mean age of 5.2 years old; standard deviation = 0.78) with typical development participated in this study. The health condition was assessed by parent-reported global child health status and the Behavior Problems Index [15,16].

### 2.2. Task

The participants were instructed to remain still in a comfortable armchair and to watch a short video presented on a laptop screen (Apple MacBook Air, (13-inch screen), Cupertino, CA, USA). The video was the Inscapes Movie [14], which has been designed and validated to increase functional connectivity and measure reliability in children.

### 2.3. fNIRS Instrumentation

**Figure 1:**
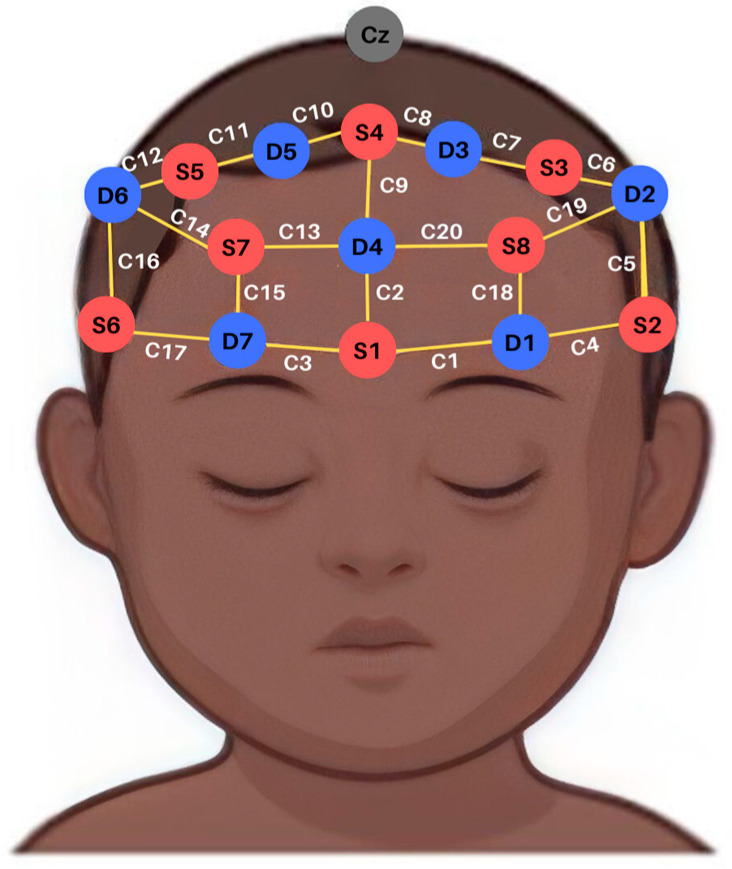
A frontal montage with 20 channels and 8 short channels (one for each detector) was used. C: channel; S: source; D: detector.

The data acquisition was performed with NIRSports (NIRx, Orlando, FL, USA) equipment comprising eight sources and eight detectors (two wavelengths, 760 and 850 nm) at a 7.81 Hz sampling rate. We use one of the eight detectors with an adapter bundle to allow short-distance channel measurements for each of the eight emitters. All optodes were located in a 20-long-distance channel layout over the prefrontal cortex using a 10-10 system cap (Easycap, Wörthsee, Germany). The montage used in this study is depicted in Figure 1.

### 2.4. Signal Preprocessing

The raw fNIRS data were pre-processed for hemodynamic state estimation given the voltage signals (using the Beer-Lambert modified equation), signals normalization to z-transform to rescale the data to wavelet denoising, motion correction using wavelet denoising (universal threshold, lambda = 0.1 to remove only spikes) [17], band-pass frequency filtering (from 0.01 to 0.08 Hz, a range similar to previous studies) [13], regression-out (multiple linear regression) of short-distance channel signals to reduce systematic and superficial artifacts [18], and normalization to variance one to rescale the data to allow multisubject group analyses. For all further analyses, only oxyhemoglobin signals were considered, since recent studies point out that oxyhemoglobin signals are more informative than deoxyhemoglobin [19,20]. Thus, to avoid a decrease in statistical power due to multiple testing corrections, we prefer to analyze only oxyhemoglobin in the illustrative application.

The signal quality was assessed at two different time points. The initial evaluation took place during the fNIRS calibration for each participant, where the power of the sources and the amplification gains of the detectors were optimized. The optodes were adjusted until the channels met the defined standards of good quality as specified by the manufacturer, which were based on the coefficient of variation and amplification gains of the signals. In the second phase, the preprocessed signals underwent visual inspection after motion correction using wavelet denoising. After careful examination, it was determined that the majority of channels from all participants achieved a satisfactory level of data quality, thereby making them suitable for further analyses.

### 2.5. A New Statistical Approach to Naturalistic fNIRS Signal Analysis

An exploratory graph-based approach was developed to identify across individuals relevant time points regarding cognitive processing. In addition, we mapped the brain regions leading to this relevance. A statistical test for the stimulus eliciting a similar hemodynamic state pattern across individuals at at least one time point and the channel was developed. If the null hypothesis is rejected in this test, we propose to conduct post-hoc exploratory analyses to further understand which time points and channels are contributing to a similar pattern while avoiding double-dipping.

For each time point, the Euclidean distance among the multivariate hemodynamic states is calculated pairwise between all participants. These distances are then used to model a network, represented by an undirected graph, and the mean closeness of such a graph is calculated across all time frames. The statistical significance is then assessed by using circular permutation testing [21], which yields a single *p*-value. If the *p*-value is less than the present Type I error, we proceed to identify which temporal frames and fNIRS channels are contributing to the result, against the null hypothesis that the stimuli do not induce common activation patterns across participants. In addition, since the proposed method is based on permutations to non-parametrically build the test statistics under the null hypothesis, It does not require data with a Gaussian distribution, and it is also suitable for the case of small samples.

The step-by-step of this proposal is described in the following:

Step (1) Between-participants distance matrix at each time-point: For each time frame, calculate the Euclidean distance of the hemodynamic states’ spatial distribution (only long-distance channels) among all participants (pairwise);

Step (2) Graph Modelling: Use this distance matrix as the adjacency matrix to model an undirected weighted graph. In this graph, each node represents a subject, and each edge is the distance between a pair of participants based on their multivariate hemodynamic state at the referred time point;

Step (3) Graph centrality: Calculate the graph’s average closeness (across nodes) corresponding to the referred time point. Closeness centrality is a well-established metric in graph analysis to quantify how similar the nodes of a network are [22]. In this case, each node is a participant;

Step (4) Test statistics: calculate the mean closeness across all time points. This is the mean statistic for this proposal;

Step (5) Statistical significance assessment: Once the observed mean closeness was obtained at the previous step, to compute the *p*-value, we needed to discover its distribution under the null hypothesis. To this end, a permutation test approach is conducted by considering 1000 circular permutations of the hemodynamic signals and recalculating the mean closeness. The number of permutations determines the level of accuracy of the empirical approximation of the null hypothesis (the second decimal place of the *p*-values is mostly the same after 1000 permutations). This approach preserves the signals’ autocorrelation and channels’ cross-correlations within the subject. However, it resamples signals under the null hypothesis that the participant’s hemodynamic states are not related to the stimuli (assuming, by the design of the experiment, that the stimulus is exactly the same for all participants). If the null hypothesis is rejected (e.g., *p*-value 5%), two additional steps are necessary to better comprehend how the stimuli are related to the changes in states. It is important to mention that this is only for descriptive purposes and not a double-dipping since the statistical significance was solely assessed in Step 5. The two additional steps are (i) the identification of the time-stamps in which the stimulus is mostly contributing to the participants’ state similarities; and (ii) the identification of the weight of each cortical region to similarities.

Step (6) Identification of the most relevant time-points: This issue might be addressed by identifying the top 5% (or any other arbitrary threshold; this parameter is solely for descriptive purposes and not statistical inference) closeness time points (see Step 3). These are the frames that are mostly contributing to the null hypothesis.

Step (7) Brain mapping: This step is carried out by repeating steps (1) to (5), removing one channel at a time, and recalculating the mean closeness. When channels of great relevance are removed, we expect that the mean closeness will be substantially reduced. In the following, for each time point, the closeness across each channel is converted to percentiles. Finally, the frequency (across) of being in these top 25% sets (the top percentile is arbitrary and solely for facilitating results interpretation and not statistical inference) is calculated for each channel, quantifying its relevance to the results obtained at Step (5).

## 3. Results

**Figure 2:**
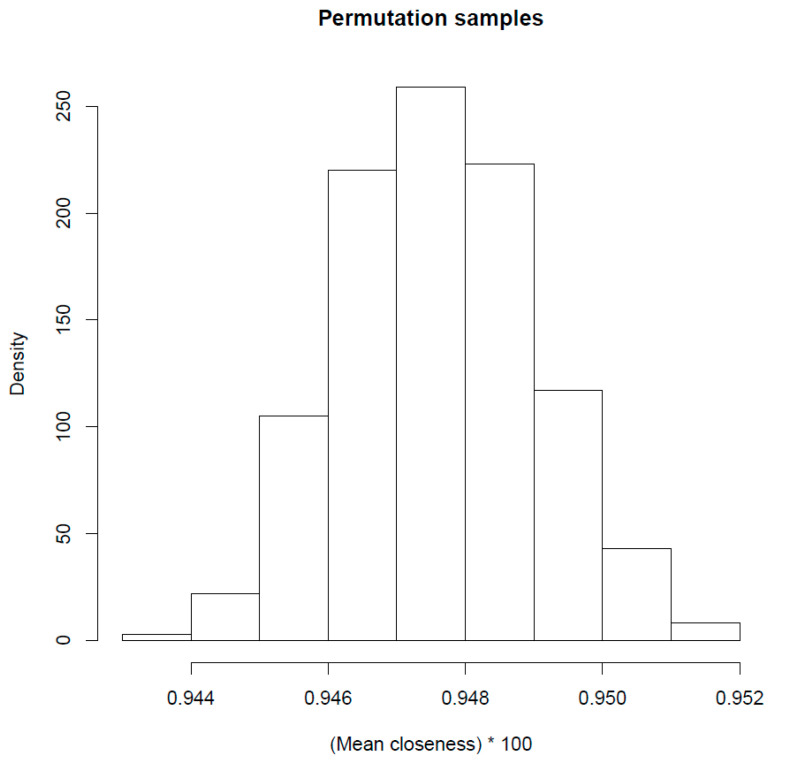
Distribution of mean closeness under the null hypothesis. The dotted line depicts the observed test statistics (*p* = 0.001).

**Figure 3:**
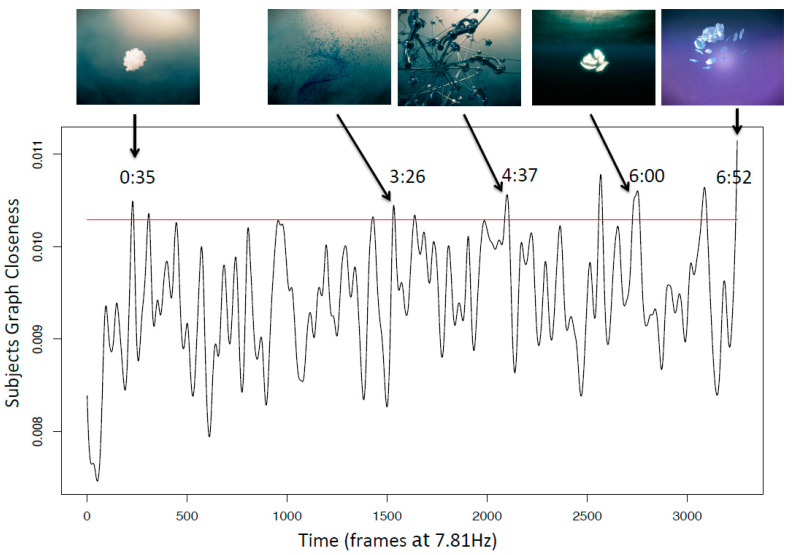
Participants Graph Closeness and 95% of frames closeness percentile (red line).

The average child BPI score was 5.2 (SD 1.7). Figure 2 depicts the observed test statistics obtained in Step (4) and also the empirically derived distribution (by circular permutation), as described in Step (5). The obtained *p*-value (*p* = 0.001) suggests that the Inscapes movie induced hemodynamic state similarities among participants at some time points. As explained in Step (6), we present the mean closeness at each frame and the top 5% percentile (red line) in Figure 3. The corrected HRF delay of 5 s timestamps (minutes: seconds) and the respective iconic representation of the visual stimuli are depicted in the same figure.

**Figure 4:**
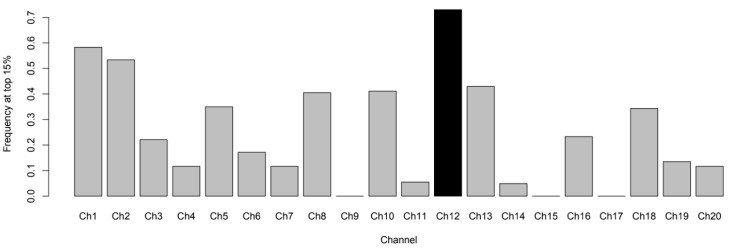
Channels’ importance regarding interbrain hemodynamic states and similarities The black bar highlights the top 5% of most contributing channels.

Finally, according to Step (7), the channels with the greatest contribution to the transitory similarity among participants are depicted in Figure 4. Channel 12 at the left dorsolateral prefrontal cortex (DLPC) was found to be among the top 25% most relevant in 73% of the frames.

## 4. Discussion

One of the main obstacles to expanding neuroimaging studies to real-life situations is the lack of proper analytical frameworks to analyze naturalistic data. Recently, methodological advances in naturalist fMRI and MEG analysis have been proposed and tested [23,24,25]. Usually, methodological advances are translated from fMRI to fNIRS analysis without explicitly taking the specific nature of the fNIRS signal into account. Despite a sparse representation of the cortical surface, the fNIRS signal has a finer temporal sampling when compared with fMRI. In the current study, we present an illustrative application for a novel analytical approach based on intersubject graph analysis to analyze the fNIRS data from children watching a movie, identifying the more inter-individually relevant scenes and also mapping the cortical regions associated with this across participants’ consistent hemodynamic responses. Importantly, this approach builds on the sampling rate of the fNIRS signal and can be applied for functional neurodevelopmental investigations.

In an illustrative application based on the Inscapes movie, we identified that the left prefrontal dorsolateral cortex (DLPFC) was similarly activated across children. Notably, the DLPFC is a component of associative networks such as the central executive network (CEN) and default mode network (DMN). Activity in this high-order associative area is more stable compared with unimodal regions [26] and also during development [27]. Moreover, Li et al. (2020) [26] have observed that fMRI signal stability decreases in the sensory cortex during movie watching and is associated with larger frontal cortex variation.

More generally, the structural and functional development of prefrontal regions is particularly protracted, with postnatal neurodevelopmental processes spanning from early childhood to adulthood [28]. Our results are consistent with this critical position of the prefrontal cortex in neurodevelopmental processes and with previous neuroimaging evidence using naturalistic stimuli. In particular, fMRI signal patterns during movie viewing have been shown to correlate with developmentally specific behavioral measures [3,29]. The identification of local and global parameters of cortical networks can be useful to elucidate important discriminative characteristics in neurodevelopment and also deviant trajectories that may lead to clinical conditions [3,30,31].

It is important to mention that this study has limitations that must be considered. First, the fNIRS data used in this study were collected exclusively from a pediatric and healthy sample. Thus, inferences derived from the present results obtained for other populations (adults and clinical populations) should be made with caution. It is recognized that changes in the central nervous system, as well as the aging process, can alter the stability of the metrics, which implies network analyses with fNIRS specifically aimed at the elderly population or with neuropsychiatric conditions. Moreover, further investigations should include peripheral measures of physiological parameters in order to better control for potential systemic physiologic artifacts on the fNIRS signal. Finally, while the current study pointed to the potential of the novel method approach to investigate naturalistic scenarios in typically developing children, our results should be treated with caution due to the small sample size and the exploratory nature of this pilot study.

In conclusion, we described a new approach to investigating spatiotemporal patterns of activity in fNIRS during movie watching and presented a proof-of-concept application in a typical sample of developing children. Methods based on the analysis of the centrality of the graph constructed from the fNIRS signal can be useful to expand the growing applicability of neuroimaging to naturalistic conditions and investigations.

## Data Availability

The data presented in this study are available on request from the corresponding author. The data are not publicly available since it was not allowed by the ethics approval.
